# From Prenatal to Preimplantation Genetic Diagnosis of β-Thalassemia. Prevention Model in 8748 Cases: 40 Years of Single Center Experience

**DOI:** 10.3390/jcm7020035

**Published:** 2018-02-20

**Authors:** Giovanni Monni, Cristina Peddes, Ambra Iuculano, Rosa Maria Ibba

**Affiliations:** Department of Prenatal and Preimplantation Genetic Diagnosis and Fetal Therapy, Microcitemico Pediatric Hospital, Cagliari 09121, Italy; cristina.peddes@gmail.com (C.P.); ambraiuculano76@gmail.com (A.I.); rosamariaibba@gmail.com (R.M.I.)

**Keywords:** β-thalassemia, prenatal diagnosis, genetic disease, chorionic villous sampling, amniocentesis, fetal blood sampling, pre-implantation genetic diagnosis, DNA, polymerase chain reaction

## Abstract

The incidence of β-thalassemia in Sardinia is high and β-39 is the most common mutation. The prevention campaign started in 1977 and was performed in a single center (Microcitemico Hospital, Cagliari, Sardinia, Italy). It was based on educational programs, population screening by hematological and molecular identification of the carriers. Prenatal and pre-implantation diagnosis was offered to couples at risk. 8564 fetal diagnosis procedures using different invasive approaches and analysis techniques were performed in the last 40 years. Trans-abdominal chorionic villous sampling was preferred due to lower complication risks and early diagnosis. Chorionic villous DNA was analyzed by PCR technique. 2138 fetuses affected by β-thalassemia were diagnosed. Women opted for termination of the pregnancy (TOP) in 98.2% of these cases. Pre-implantation genetic diagnosis (PGD) was proposed to couples at risk to avoid TOP. A total of 184 PGD were performed. Initially, the procedure was exclusively offered to infertile couples, according to the law in force. The success rate of pregnancies increased from 11.1% to 30.8% when, crucial law changes were enacted, and PGD was offered to fertile women as well. Forty years of β-thalassemia prevention programs in Sardinia have demonstrated the important decrease of this severe genetic disorder.

## 1. Introduction

β-thalassemia is a common inherited autosomal recessive defect characterized by severe microcytosis, hepatosplenomegaly and other particular skeletal malformations such as dysmorphic facies and osteoporosis. Without treatment, affected children fail to thrive and their life expectancy is shortened [1].

The genetic disorder results from reduced (β+) or absent (β°) production of the β-globinchains [2,3].

This genetic disease is very frequent in the Mediterranean area (Italy, Greece and Turkey in particular), North Africa, the Middle East as well as in the Far East and East Asia [4]. β-thalassemia has spread around the world through the immigration of populations. It is estimated that 1.5% of the world’s population for a total of 90,000,000 people are carriers: about 400,000 of them are affected and each year there are about 60,000 new cases.

In Sardinia, an Italian island in the Mediterranean Sea with a population of 1.7 million, the carrier frequency incidence is about 12% with one couple out of 60 at risk of genetic transmission of disease and each year in Sardinia, the possibility to have a newborn affected by thalassemia is 1 every 250 newborns [5,6].

Regular blood transfusion and iron chelation with desferrioxamine B is the current therapy adopted for the survival and improvement of the quality of life. Nowadays, the only possibility of healing is marrow transplantation of allele compatible human leukocyte antigen (HLA), with a success rate of 90% [7]. Gene therapy is experimental, but it is emerging as a powerful approach.

The only prevention strategy of this severe genetic disease has been for many decades the identification of the carrier and the reduction of affected fetuses.

In this paper, we report our experience in β-thalassemia prevention carried out from 1977 to 2017 in 8748 cases (8564 fetuses and 184 embryos), performed by prenatal and pre-implantation genetic diagnosis in a single center, the Microcitemico Pediatric Hospital in Cagliari.

## 2. Carrier Screening

β-thalassemia is a condition determined by heterogeneous molecular anomalies. More than 200 different molecular defects are defined and 95% are caused by β-globin gene point mutations [8,9].

The most common mutations in Italy are: c.118C>T (β°39), c.93-21G>A, c.92+6T>C and c.315+1G>C,A,T [5]. Mutation c.118C>T is the most common point mutation in Sardinia (95.7%) and it was described for the first time in 1981 by our group (Table 1) [10,11].

The voluntary screening program started in Sardinia in the 1970s and it was based on extensive information and educational activities. The program involved all mass media, family doctors, obstetricians, pediatricians, midwives; topic-related brochures and leaflets were distributed among the population [12].

At the beginning, our prenatal screening program was aimed at parents of β-thalassemic progeny, who were offered PGD and invasive prenatal diagnosis.

Population screening was performed by offering free hematological tests to identify β-thalassemia carriers and genetic mutations [12].

Genetic counseling on β-thalassemia, genetic mutations, reproductive options, PGD as well as the efficacy, accuracy and risks of fetal diagnosis procedures was offered to all couples of healthy carriers. Additional neonatal therapy options were provided. HLA typing on embryo-fetal DNA was proposed to evaluate the option of bone marrow transplantation to newborns [13]. After genetic counseling, 97% of pregnant and 93% of non-pregnant women accepted antenatal invasivediagnosis [1].

Identifying β-thalassemia genetic mutations in the affected fetus was essential not only for the expectant couple but for all family members to whom further hematological profile analysis was suggested.

The method of DNA amplification through PCR is the basis of all techniques used for the diagnosis of genetic defects responsible for β-thalassemia [14].

The most common procedures such as amplification refractory mutation system (ARMS) and reverse dot-blot analysis (RDB) identify the mutations which are already known by using specific primers [15,16].

Different laboratory kits are available for searching for the most frequent ethnic β-gene point mutations. In our screening program, we used the Mediterranean mutations kit.

In addition, CGH-Array can be used for identifying deletions of the β-globin gene. These arrays use probes spaced at short intervals throughout the locus to finely map the deletion breakpoints, to design PCR primers to amplify the breakpoint region and to determine the sequences flanking the breakpoints. This technique is employed to confirm the breakpoints for several known mutations causing δβ-thalassemia and hereditary persistence of fetal hemoglobin (HPFH) as well as previously unmapped deletions [17,18,19,20]. In such cases, multiplex ligation-dependent amplification (MLPA) may be used to determine the presence of an unidentified α- or β-globin gene deletion, by assessing DNA copy number changes. It is an efficient tool to detect β-globin locus control region (β-LCR) deletions combined with long-range PCR and DNA sequencing to pinpoint deletion breakpoints [21]. We did not use the CGH-array in our screening program because we identified mainly the most frequent Mediterranean β-gene mutations.

## 3. Fetal and Embryo Invasive Procedures

In the last 40 years we performed 8564 fetal diagnoses and 184 PGD procedures using different invasive approaches and analysis techniques. The sampling success rate was very high, the fetal loss rate and misdiagnosis rates were consistently low and progressively even lower. The percentage of women who chose invasive prenatal diagnosis after genetic counseling was high during the whole 40-year period (Table 2).

### 3.1. Prenatal Diagnosis

We started to perform prenatal diagnosis of β-thalassemia in 1977, by placentocentesis (sampling of fetal blood from the placenta) at 20 weeks of gestation [22]. We obtained an amount of fetal blood adequate for analysis in 99% of the cases. Fetal mortality associated with placental aspiration was 6.1%. The molecular analysis provided reliable results [23].

Since 1983–1985 we performed fetal blood sampling after 18 weeks by fetoscopy by cordocentesis. Cardiocentesis or hepatic vein puncture were performed only in selected cases when other techniques failed or were not found suitable [24]. The success rate of these procedures was 99% and the risk of complications was lower than that of placentocentesis; fetal miscarriage risk was2% [25].

In 1982, the advances in the field of molecular biology research [26,27] gave us the possibility to use fetal DNA extracted from amniotic fluid at 16–18 weeks. In 1983, thanks to the introduction of trans-cervical chorionic villous sampling (TC-CVS), prenatal diagnosis shifted to the first trimester of pregnancy [28].

From 1985 until today, the only invasive diagnosis procedure employed in our center was trans-abdominal chorionic villous sampling (TA-CVS). This technique can be performed free-hand or with needle guide. Free-hand technique is considered the golden standard technique. It is performed with a single 20-gauge spinal needle that can be inserted perpendicularly to the chorion or tangentially to the ultrasound scanner. The procedure has to be performed under continuous ultrasound guidance. Up-and-down movement must be made to cut the villi and negative pressure on the syringe has to be applied to aspirate chorionic tissue [29].

The TA-CVS free-hand technique is to be preferred due to lower fetal loss risk of and lower incidence of vaginal bleeding compared to TC-CVS [30]. The risk of infections is lower when the procedure is performed trans-abdominally [25].

Fetal loss is the most important complication of invasive procedures; according to a recent meta-analysis, procedure related risk is strictly linked to the operator experience. Fetal loss risk is lower when the procedure is performed in centers of proven experience [31].

CVS is generally performed between 10 and 12 weeks of gestation, in the same period as the fetal nuchal translucency (NT) measurement and the biochemical combined screening. CVS samples before the 10th week seemed to be associated with a higher risk of abortion and fetal complications such as transverse limb defects [32,33,34].

All patients underwent genetic counseling before the invasive prenatal diagnosis procedure. Maternal age >35 and/or high risk combined test and/or enlarged NT and/or alterated biochemical test were indications for studying the fetal karyotype. Currently, we propose TA-CVS for β-thalassemia analysis after combined test screening. This is the best screening strategy to avoid a second sampling procedure [35].

When patients were referred to our center after 13 weeks of gestation, we proposed TA-CVS by 19 or 18-gauge spinal needle rather than amniocentesis [36,37]. TA-CVS can be performed from 13 weeks to the end of pregnancy.

In twin pregnancy a careful ultrasound check-up was performed in order to choose the best diagnostic approach and to reduce the risk of erroneous attribution of the sample. Chorionicity, fetal labeling, fetal biometry, discordant sex and discordant ultrasound fetal anomaly were described before the procedure [38]. The sampling of both fetuses was necessary in dichorionic pregnancies, while in monochorionic a single sample was enough to check monogenic diseases. In dichorionic pregnancies, two separate samplings were performed one at each trophoblastic area. It was possible to perform a single insertion to sample the two placentae when the two chorions were contiguous, although the risk of contamination was higher. To reduce the risk of inaccurate results, the sampling spot was chosen near the cord insertion, thus avoiding the area around the dividing membrane [39]. If one of the two fetuses were affected we proposed selective feticide [40].

Until December 2017 we carried out 8564 fetal diagnosis procedures of β-thalassemia and 2138 fetuses were affected. In 98.2% of these cases, women took the painful decision to voluntarily interrupt the pregnancy. Our pediatric hospital has become an important and efficient center for child therapy and bone marrow transplantation in order to limit termination of pregnancy (TOP) in case of β-thalassemia-affected fetuses (Table 3).

### 3.2. Embryo Analysis by PGD

PGD studies DNA obtained by embryonic cells by analyzing blastomeres or trophectoderm cells to test the mutations in the target gene before transferring the embryo in utero. PGD allows high-risk couples to avoid TOP. It is an extremely useful technique for patients who would never consider TOP as a choice for ethical and religious issues or those who give up a pregnancy a priori because of the existing high risk to conceive an affected fetus [41].

Several studies have shown that couples who opt for a voluntary TOP, after invasive prenatal diagnosis and above all after a previous TOP, prefer PGD rather than resorting to CVS again [42].

PGD requires in vitro fertilization (IVF) techniques, intracytoplasmatic sperm injection (ICSI) and hormonal stimulation [43].

In our experience, multidisciplinary approach including appropriate genetic counseling, referral to both a fertility center and to a highly specialized molecular genetics laboratory was mandatory. Counseling of couples considering PGD offered information regarding the genetic and reproductive status, the risks associated with IVF procedures and with embryo biopsy, the technical limitations of DNA analysis, including the risk of failure of the procedure as well as that of misdiagnosis. There was also the possibility that no embryos were fit for transfer if they were abnormal or if the fertilization failed. The management and the cryoconservation of those embryos that were not transferred also had to be considered [41].

Cleavage stage biopsy was performed on the third day after insemination, and on the fifth day by trophectoderm cells. Only embryos not affected by thalassemia were then selected and transferred in utero.

The success rate of the PGD cycle is strictly linked to the efficiency and accuracy of each stage. Maternal age, stimulation protocol, number of cumulus complex oocytes, embryo culture, embryo biopsy and molecular diagnosis could influence its success [44].

PGD, at the beginning of our experience, was applied only to infertile patients with 11.1% of pregnancy rate per embryo transfer. Subsequently, when fertile patients were included, this rate increased at 30.8% pregnancies per embryo-transfer [44].

We started PGD in 2002 and performed 42 cases using one or two blastomeres and DNA analysis until 2004. In 2004, a law on assisted fertilization which prohibited PGD was passed so it was not possible to perform PGD from 2004 to 2014 [45]. Recently, numerous Courts among which the Cagliari Civil Court and the Constitutional Supreme Court in Italy mentioned the right to PGD on the basis of Law 194 of 1978 about voluntary interruption of pregnancy declaring that “the selection of embryos is not a crime, even in cases where this is exclusively aimed at avoiding the implantation, in the uterus of the woman, of embryos suffering from genetic transmission diseases that meet the criteria of gravity”.

Since 2014 we performed 184 procedures using mainly biopsy by blastomeres and, more rarely, by trophectoderm cells (Table 4).

PGD may be performed on one or two biopsied blastomeres from a single embryo. Performing two-cells genetic testing guarantees higher accuracy, but it is less favorable for pregnancyoutcome [46], so we opted for single-cell genetic test. The major disadvantage of cleavage stage biopsy is the limited amount of genetic material.

During trophoectoderm biopsy, 5–10 cells can be sampled so, more genetic material is available for genetic analysis. Trophoectoderm cells develop to form placenta and other extraembryonic tissue so blastocyst biopsy is considered a safe technique. Unfortunately, the time available to complete the diagnosis before cryopreserving the embryos is limited and only about 40–50% of pre-implantation embryos develop at this stage in vitro [47].

We investigated HLA compatibility in some cases of β-thalassemic fetuses. In the fetuses where compatibility with a close family member was found, we proposed the continuation of pregnancy and possible bone marrow transplantation after birth [48]. In our experience we have been asked to perform HLA typing of an unaffected fetus and to perform bone marrow transplantation on affected siblings. In these cases, umbilical cord stem cells were collected and stored, where possible, for a transplantation [13].

### 3.3. Molecular Analysis Techniques

The molecular biology techniques employed for the diagnosis of β-thalassemia have changed over time in relation to the type of material taken and the development of techniques.

At the beginning of our experience, the analysis was performed on fetal blood taken by placentocentesis. Currently, we use the technique of globin chain synthesis analysis by column chromatography on fetal blood obtained by placental aspiration. In 99% of sampling we obtained sufficient fetal blood to perform the analysis. The biochemical analysis gave reliable results. We had two misdiagnoses (0.2%): one due to a non-globin protein comigrating with the β-chains and the other for a misclassification of the type of thalassemia in the family [23].

Fetal DNA for analysis was obtained from either amniotic liquid or chorionic villi, but, as described, CVS was the procedure of choice. The CVS provides a source of high-quality DNA in more than sufficient quantity to complete the prenatal DNA analysis [49,50,51].

Almost all methods for DNA analysis of hemoglobimopathies currently in use are based on PCR. There are many different PCR-based types of analysis. The laboratory techniques have to be chosen on the type and variety of the variants likely to be encountered in the individuals (population group) tested and not on the basis of technical expertise [50].

For the Sardinian ethnic group mutation, our center laboratory employed the ARMS technique in combination with the RDB analysis. Knowing the molecular defect present in both parents was mandatory before performing prenatal diagnosis procedures [27].

Chorionic villus DNA had to be analyzed with primer-specific amplification (ARMS), using separate pairs of primer. When the spectrum of mutations to be searched was complex, ARMS was not the most appropriate method. In these cases, RDB was more informative and efficient, it was able to screen simultaneously a large number of mutations. If performed by expert hands, primer-specific amplification was safe and particularly useful in fetal DNA analysis to search for mutations previously detected in the parents [16,52]. The combination of ARMS and RBD technique reduced the risk of misdiagnosis.

The main causes that could lead to an incorrect diagnosis were inability to amplify the fragment of target DNA, mispaternity, maternal contamination or sample exchange. Performing the analysis of two different chorionic villus fragments and the execution of the examination by two different operators was useful to reduce the risks.

Furthermore, to reduce the risk of misdiagnosis it was strongly recommended to include maternal cell contamination test and careful dissection of maternal decidua. In our laboratory variable number tandem repeat (VNTR) was performed to exclude contamination above all in maternal decidua [53].

At the beginning of our experience we registered one incorrect laboratory diagnosis performed on DNA extracted through TC-CVS; the employed analysis was enzymatically restricted genomic DNA by allele-specific oligonucleotide radioactive probes [27]. After 1985 we introduced PCR by RDB, allelic-specific oligonucleotide primers by ARMS. So far, we have not had any misdiagnosis by TA-CVS using also only a few amplification cycles and two different overlapping amplified DNA fragments in duplicate analyses [54].

Molecular methods for mutation detection in PGD have always been based on multiple steps of PCR. Multiplex PCR was used to amplify both the region of β-globin gene, subsequently two nested PCR reactions was applied to produce DNA fragments suitable for the analysis. The presence of β-globin gene mutations was identified by a subsequent minisequencing reaction [43,44,55]. There are many factors that make the PGD protocols difficult to develop and apply. Small amount of genetic material is available for analysis and therefore the results can be non-diagnostic, in particular if on single-cell is performed. Obtaining a diagnostic result can be subject to several other drawbacks: the high likelihood of complete amplification failure; the possibility that one of the two targets alleles fails to amplify at least to detectable levels (known as allelic dropout; ADO), that can lead to false negative results; and the possibility of sample contamination [56].

## 4. Discussion

Our results demonstrate the permanent efficacy of the β-thalassemia continuous prevention policy by screening, embryo and fetal diagnosis in Sardinia and this model can be exported and applied successfully anywhere in the world. Fellows from all over the world have been trained in invasive prenatal diagnosis procedures at our center [57,58].

The positive response after genetic counseling regarding the possibilities for screening and antenatal diagnosis was very high and the whole Sardinian population collaborated very actively. The close collaboration between molecular biologists, genetists, obstetricians and pediatricians available in our center was another important factor for the success of this prevention model in terms of efficacy, safety and accuracy of diagnosis [49]. Embryo and fetal sampling procedures safety improved at the same pace as molecular techniques. Shifting prenatal diagnosis of thalassemia from the second trimester to the first trimester of pregnancy was thus made possible [59]. As a consequence, the number of women who decided to undergo invasive procedures increased significantly and even more so with the introduction of PGD, which enriched further the β-thalassemia prevention strategies [42] (Table 5).

The low risk of fetal loss associated with TA-CVS and the feasibility of HLA typing techniques led to a further increase in reproductive choices, making prenatal diagnosis even more ethically accepted. In one case we also performed fetal bone marrow transplantation in utero, in the first trimester of pregnancy, but it was unsuccessful [60].

**Figure 1:**
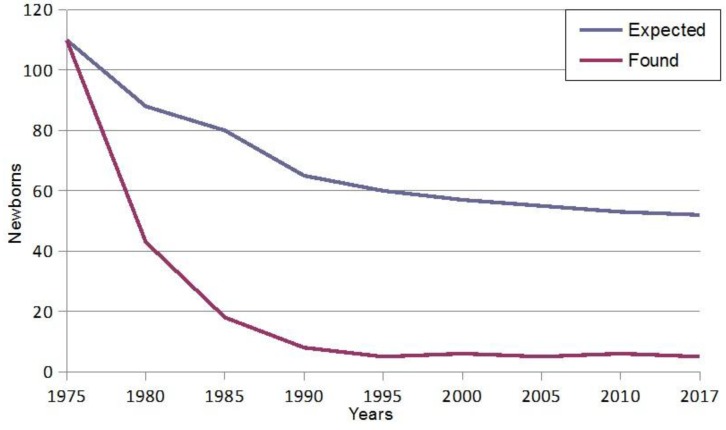
Fall in the birth rate of homozygous β-thalassemia in Sardinia.

Figure 1 shows that thanks to the forty years of continuous prevention programs of β-thalassemia in Sardinia we witness a strong decrease in the number of affected fetuses [49].

In recent decades, important research such as non-invasive prenatal testing (NIPT) using cell-free DNA in early gestational age has been developed for the diagnosis of aneuploidies. NIPT for all genetic conditions is likely to become feasible in the future as technological advances are made and costs of sequencing decreases [61].

Digital PCR, with the approach known as relative mutation dosage (RMD), based on quantification of normal and/or mutated alleles present in the cell free fetal DNA (cff-DNA) represented the first technology proposed to non-invasive prenatal assessment of monogenic diseases such as thalassemia [62,63].

With the introduction of next generation sequencing (NGS), several new approaches have been developed for non-invasive prenatal assessment of thalassemia but they are still not effective diagnostic methods [64,65].

Thanks to the incessant development of new technologies, it will soon be possible to perform non-invasive prenatal diagnosis of monogenic diseases. The evolution of gene therapy raises many hopes [66,67].

## 5. Conclusions

In 1977, when prevention strategies were not yet available in Sardinia, there were 120 β-thalassemic newborns, compared to only 3–5 affected newborns nowadays due to the enormous advance in the field of antenatal diagnosis. The birth of β-thalassemia-affected babies is determined by false paternity, decline of antenatal testing, and avoiding of TOP for religious or ethical reasons.

## Figures and Tables

**Table 1:** Frequency of β-thalassemia mutations in Sardinia.

Mutation	International Name (HGVS)	Frequency (%)
β-39	c.118C>T	95.7
β-6	c.20delA	2.2
β-76	c.230delC	0.7
β I-110	c.93-21G>A	0.5
β II-745	c.316-106C>G	0.4
β-87	c.-137C>A,G,T	0.2
β I-6	c.92+6T>C	0.2
β II -1	c.315+1G>C,A,T	0.1
β I -1	c.92+1G>T,A	0.03

**Table 2:** Invasive prenatal diagnoses of β-thalassemia in Sardinia: 8564 in 40 years (1977–2017).

Techniques	No.	Years	Gestational Age (Weeks)	Failure No.	Fetal loss (%)	Misdiagnosis
Placentacentesis	981	1977–1983	18–24	10	5.2	2
Fetoscopy	67	1983–1985	18–24	2	5.6	-
Cordocentesis	120	1984–1985	18–24	1	2.1	-
Hepatic vein puncture	3	1984–1986	18–24	-	-	-
Cardiocentesis	3	1984–1986	18–24	-	-	-
Amniocentesis	203	1982–1983	16–18	6 ^2^	2.6	-
Trans-cervical CVS ^1^	572	1983–1986	9–13	1 ^2^	4.2	1
Trans-abdominal CVS ^1^	6615	1985–1917	6–24	-	0.6	-

^1^ CVS, chorionic villus sampling; ^2^ Due to limited amount of DNA at sampling.

**Table 3:** Fetal diagnosis of β-thalassemia 1977–2017.

	No.
Women	8564
Normal fetuses	2141
Healthy carriers	4285
Affected fetuses	2138 ^1^

^1^ 98.2% women opted for termination of pregnancy.

**Table 4:** Embryo procedures—pre-implantation genetic diagnosis.

	No.
Women	184
Age	33.9 ± 5.8
Cycles	223
Stage of biopsy 3 (blastomere)	195
Stage of biopsy 5 (trophectoderm cells)	28
Embryos transferred	140
Miscarriages	7 ^1^
Clinical pregnancies	38

^1^ All in the first trimester (5–12 weeks).

**Table 5:** Acceptance of prenatal diagnosis of β-thalassemia according to the invasive procedures.

Technique	Acceptance (%)
Fetal blood sampling	93.2
Amniocentesis	96.4
Chorionic villus sampling	99.3

Based on published parameters [59].
